# Inhibition of PP2A by LIS1 increases HIV-1 gene expression

**DOI:** 10.1186/1742-4690-3-65

**Published:** 2006-10-02

**Authors:** Nicolas Epie, Tatyana Ammosova, Willie Turner, Sergei Nekhai

**Affiliations:** 1 Center for Sickle Cell Disease, Howard University College of Medicine, 520 W Street N.W., Washington, DC 20059, USA; 2 Department of Microbiology, Howard University College of Medicine, 520 W Street N.W., Washington, DC 20059, USA; 3 Department of Biochemistry and Molecular Biology, Howard University College of Medicine, 520 W Street N.W., Washington, DC 20059, USA

## Abstract

**Background:**

Lissencephaly is a severe brain malformation in part caused by mutations in the LIS1 gene. LIS1 interacts with microtubule-associated proteins, and enhances transport of microtubule fragments. Previously we showed that LIS1 interacts with HIV-1 Tat protein and that this interaction was mediated by WD40 domains of LIS1. In the present study, we analyze the effect of LIS1 on Tat-mediated transcription of HIV-1 LTR.

**Results:**

Tat-mediated HIV-1 transcription was upregulated in 293 cells transfected with LIS1 expression vector. The WD5 but not the N-terminal domain of LIS1 increases Tat-dependent HIV-1 transcription. The effect of LIS1 was similar to the effect of okadaic acid, an inhibitor of protein phosphatase 2A (PP2A). We then analyzed the effect of LIS1 on the activity of PP2A *in vitro*. We show that LIS1 and its isolated WD5 domain but not the N-terminal domain of LIS1 blocks PP2A activity.

**Conclusion:**

Our results show that inhibition of PP2A by LIS1 induces HIV-1 transcription. Our results also point to a possibility that LIS1 might function in the cells as a yet unrecognized regulatory subunit of PP2A.

## Background

Tat protein is a transcriptional activator encoded in the genome of HIV-1 (reviewed in [1]). Tat binds to a transactivation response (TAR) RNA [1] and activates HIV-1 transcription by recruiting transcriptional co-activators that include Positive Transcription Elongation Factor b and histone acetyl transferases [2-4]. In addition to its function in HIV-1 transcription, Tat also interacts with a number of cellular factors thus affecting host cellular functions [5,6]. In T cells, Tat causes apoptosis by binding to microtubules and affecting microtubule formation [7]. Tat also causes apoptosis in neurons apparently by changing polarity of the neuronal membranes [8,9]. Previously, we reported that Tat binds to LIS1 [10]. LIS1 is a microtubule binding protein and its mutation causes Lissencephaly, a severe brain malformation [11]. Lissencephaly is caused by abnormal neuronal migration during brain development [12]. LIS1 is 45 kD protein that contains seven WD repeats and an N terminal domain devoid of the repeats. The WD repeats-containing proteins fold into a beta propeller structure that participates in protein-protein interaction in cells [13]. The diverse family of WD40 proteins includes B-subunits of protein phosphatase 2A (PP2A). PP2A is a major serine/threonine phosphatase found mainly in the nucleus but also present in the cytoplasm [14]. PP2A catalytic subunit associates with the A subunit to form the core enzyme, and with the A and B subunits to form the holoenzyme [15]. The B subunits are diversified and represented by a variety of proteins ranging from 45 kD to 55 kD [15-17]. B subunits target PP2A to different locations within the cell [18-20]. PP2A was reported to affect HIV-1 transcription both positively and negatively. Deregulation of cellular enzymatic activity of PP2A inhibited Tat-induced HIV-1 transcription [21,22]. Expression of the catalytic subunit of PP2A enhanced activation of HIV-1 promoter by phorbol myristate acetate (PMA), whereas inhibition of PP2A by okadaic acid and by fostriecin prevented activation of HIV-1 promoter [22]. In contrast, inhibition of PP2A was shown to induce phosphorylation of Sp1 and upregulate HIV-1 transcription [23].

In this report, we investigate the effect of LIS1, full length or its isolated domains, on Tat mediated HIV-1 transcription in 293 cells. We compared the effect of LIS1 with the effect of okadaic acid, a known inhibitor of PP2A. We also analyzed the effect of LIS1 on strong viral cytomegalovirus (CMV) promoter and a strong cellular phosphoglycerate kinase (PGK) promoter. Observing similar effects of LIS1 and okadaic acid, we also analyzed the effect of LIS1 on the activity of PP2A *in vitro*. Our results presented here point to LIS1 as a yet unrecognized regulator of PP2A that may contribute to the regulation of HIV-1 transcription.

## Results

### LIS1 induces HIV-1 transcription

**Figure 1:**
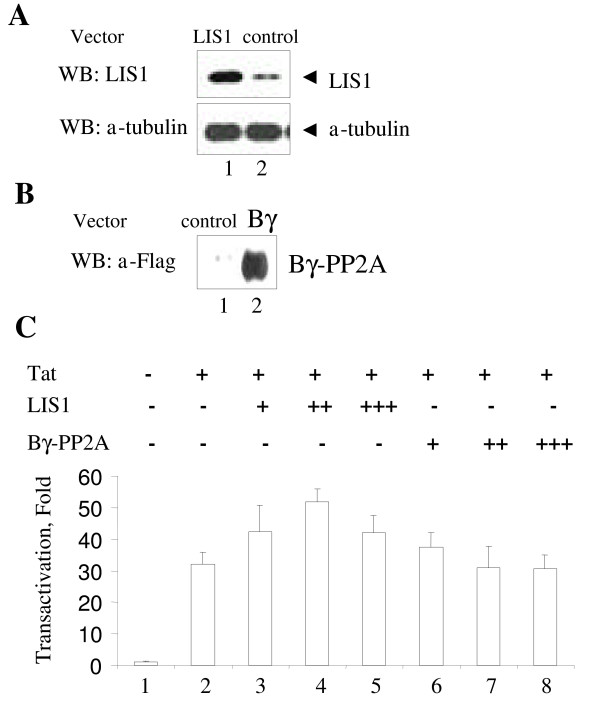
**LIS1 induces HIV-1 transcription**. ***A ***and ***B***, 293 cells grown in DMEM to 50% confluency were transfected with a LIS1 expression vector (panel A, lane 1), Flag-Bγ expression vector (panel B, lane 2) or pCI expression vector. Cells were lysed in SDS-loading buffer. Lysates were resolved on 12% SDS PAGE followed by immunoblotting with anti-LIS1, anti-α-tubulin or anti-Flag antibodies as indicated. ***C***, 293 cells were grown to 50% confluency and transfected with different concentrations of vectors expressing LIS1 (lanes 3–5) or Bγ subunit of PP2A (lanes 6–8) combined with HIV-1 LTR *lacZ *and Tat expression vectors. The pCI-neo vector was added to keep constant the amount of CMV promoter-containing pCI vector in the transfection. Lane 1, control transfected with only HIV-1 LTR-*LacZ*. Lane 2, control transfected with HIV-1 LTR-*LacZ *and Tat expression vectors. Expression of β-galactosidase was analyzed using ONPG-based assay. The results are expressed as a fold of transactivation.

**Figure 2:**
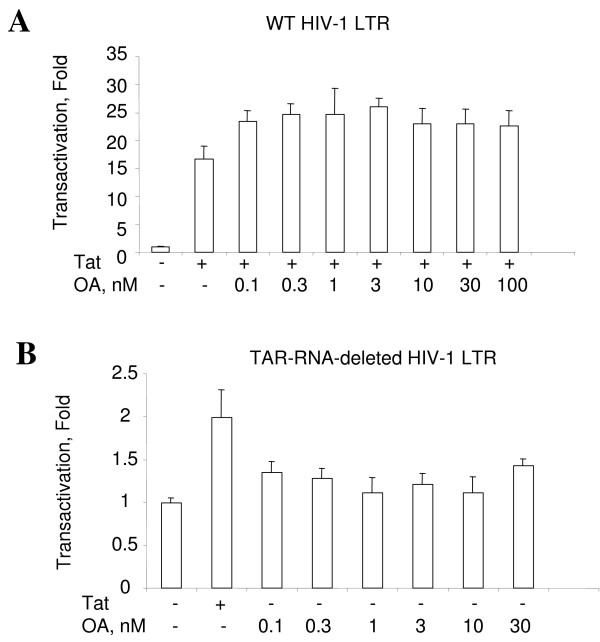
**Upregulation of HIV-1 transcription by okadaic acid**. 293 cells were grown to 50% confluency and transfected with a combination of HIV-1 LTR-*LacZ *and Tat expression vectors **(panel A) **or TAR deleted mutant of HIV-1 LTR-*LacZ *expression vector **(panel B)**. Okadaic acid was added in increasing concentrations and the cells were assayed for β-galactosidase at 48 hours posttransfection. The results are expressed as a fold of transactivation.

We analyzed the effect of LIS1 overexpression on HIV-1 transcription in 293 cells. Protein level of LIS1 was elevated in the cells transfected with LIS1-expressing vector as compared to the control cells transfected with the empty vector (Fig. 1, panel A lanes 1 and 2). Immunoblotting of tubulin was used as a control for equal protein load (Fig. 1, panel A). We also expressed a Flag-tagged Bγ-subunit of PP2A (Bγ) [24] and its expression was verified by immunoblotting with anti-Flag antibodies (Fig. 1, panel B, lane 2). Co-transfection of LIS1 expression vector with HIV-1 LTR-*Lac Z *and Tat-expression vectors increased Tat-induced transcription in 293 cells (Fig. 1, panel C, compare lanes 3–5 to lane 2). In contrast, co-transfection with the Bγ subunit of PP2A, which also contains WD40 repeats, did not increase Tat mediated HIV-1 transcription (Fig. 1, panel C, lanes 6 to 8). Although expression of the Bγ did not have an effect on Tat-induced transcription, we argue that LIS1, a WD40 protein having a structural and amino acid sequence similarity to the PP2A regulatory B-subunit, might still function as a modulator of cellular PP2A. Thus we compared the effect of LIS1 on HIV-1 transcription with the effect of okadaic acid. Okadaic acid specifically inhibits PP2A at low concentration (0.1 – 1 nM) and inhibits both PP1 and PP2A at higher concentration (0.1–1 μM) [25]. Okadaic acid treatment of 293 cells transfected with HIV-1 LTR-*Lac Z *and Tat expression vectors showed increase in Tat-induced transcription (Fig. 2, panel A). In contrast, okadaic acid had no effect on the expression of the TAR RNA-deleted HIV-1 LTR-*LacZ *(Fig. 2, panel B). Thus taken together, these results show that in 293 cells both LIS1 and okadaic acid upregulate HIV-1 transcription.

### WD5 domain of LIS1 upregulates Tat mediated transcription

**Figure 3:**
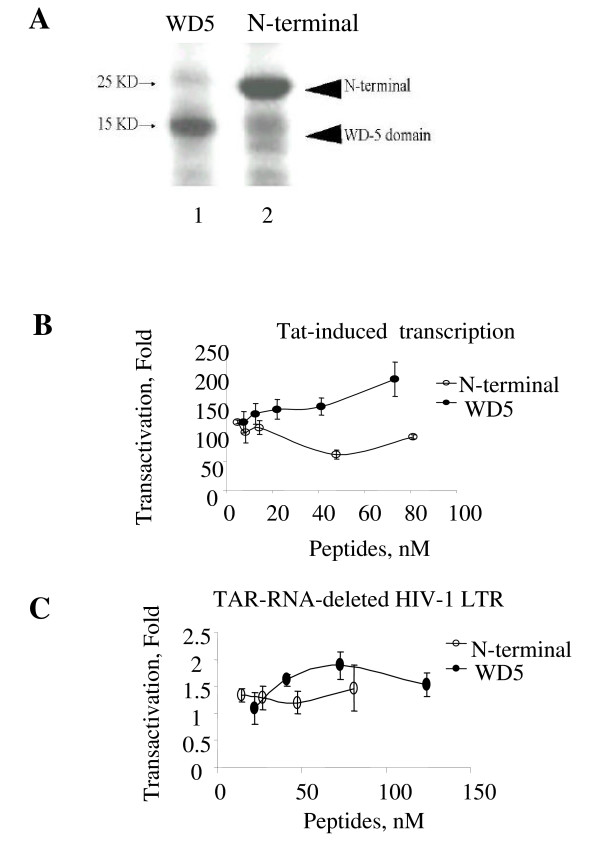
**WD5 domain of LIS1 upregulates Tat mediated HIV-1 transcription**. ***A***. The WD5 domain and N-terminal domain of LIS1 were expressed in *E. coli *and extracted from the inclusion bodies as described in Methods. The dialyzed peptides were resolved on 12% SDS-PAGE gel and stained by Coumassie blue. ***B***. 293 cells transfected with HIV-1 LTR-*LacZ *and Tat expression vectors and treated at 24 hrs posttransfection with WD5 domain or the N-terminal domain of LIS1. ***C***, 293 cells transfected with TAR RNA-deleted HIV-1 LTR-*LacZ *vector and treated as in panel A. The results are presented as a fold of transactivation.

**Figure 4:**
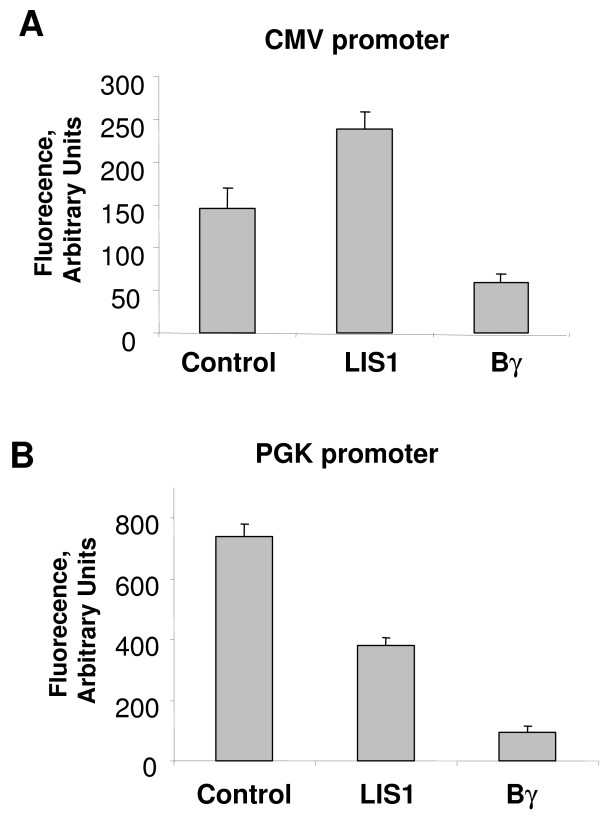
293T cells were grown to 50% confluency and transfected with vectors expressing EGFP under the control of CMV (panel A) or PGK (panel B) promoters without or with vectors expressing LIS1 or Bγ subunit of PP2A. The EGFP expression was measured by fluorescence in the cellular lysates at 480 nm excitation and 510 nm emission as described in Methods.

Next we analyzed whether a particular region of LIS1 was responsible for the increase of HIV-1 transcription. Our previous study indicated that Tat interacts with WD5 domain of LIS1 but not with the N-terminal portion of LIS1, which is devoid of the WD40 domains [10]. WD5 and the N terminal domain of LIS1 were expressed in bacteria as fusions with homeodomain-derived cell penetrating peptide to allow uptake of the fused LIS1 domains into the mammalian cells. The expression of WD5 and N-terminal domain of LIS1 was verified by SDS PAGE (Fig. 3A). 293 cells were transfected with HIV-1 LTR-*lacZ *and Tat-expression vectors and the transfected cells were treated with the cell permeable peptides for 24 hrs following the transfection. Treatment of the transfected cells with WD5 peptide increased Tat-induced transcription (Fig. 3B). In contrast, treatment with the peptide containing N-terminal domain of LIS1 showed no effect on Tat-transactivation (Fig. 3B). The peptides did not have a profound effect on the basal HIV-1 transcription from LTR containing a TAR deletion (Fig. 3C). Taken together, these results suggest that WD5 domain of LIS1 might be responsible for the induction of HIV-1 transcription. To determine whether the effect of LIS1 on the HIV-1 promoter was specific, we transfected 293T cells with vectors expressing EGFP under the control of viral cytomegalovirus (CMV) or cellular phosphoglycerate kinase (PGK) promoters. LIS1 induces transcription from CMV promoter (Fig. 4A) but inhibited transcription from PGK promoter (Fig 4B). In contrast, expression of Bγ inhibited both CMV and PGK-mediated transcription (Fig. 4).

### The WD5 domain of LIS1 inhibits phosphorylase-phosphatase activity of PP2A

**Figure 5:**
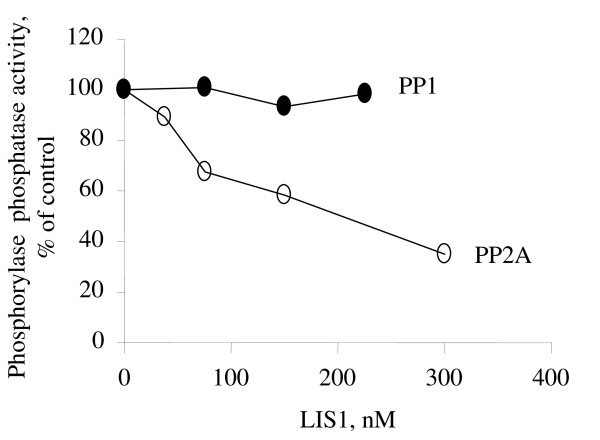
**LIS1 inhibits PP2A activity *in vitro***. Phosphatase assay was performed as described in Methods. Phosphorylase-*a *substrate, PP1 or PP2A were incubated with indicated concentrations of LIS1 protein. Results are presented as a percent of untreated control

**Figure 6:**
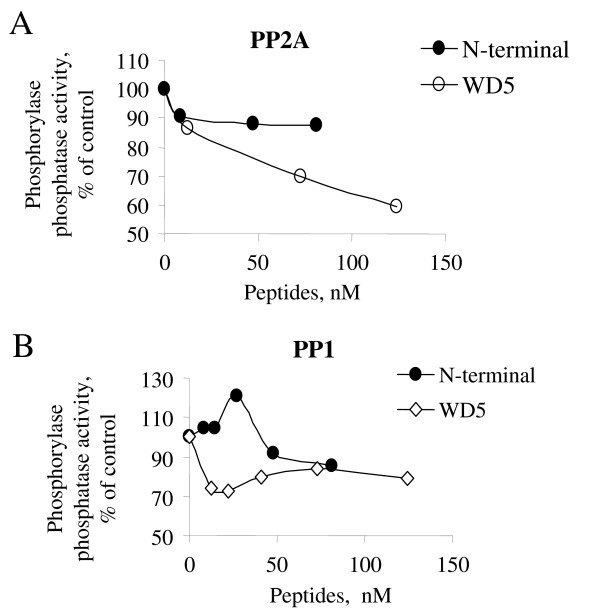
**WD5 of LIS1 inhibits PP2A activity *invitro***. Phosphatase assay was performed as described in Methods. Phosphorylase-*a *substrate, PP2A (panel ***A***) or PP1 (panel ***B***) were incubated with indicated concentrations of WD5 or N-terminal peptides. Results are presented as a percent of untreated control

To determine whether the effect of LIS1 is due to the inhibition PP2A, we analyzed the effect of LIS1 on the phosphorylase phosphatase activity of PP2A. Glycogen phosphorylase-*a*, a general substrate of PP2A and PP1 phosphatases was prepared by phosphorylating phosphorylase-*b *with phosphorylase kinase using (γ^32^P) ATP [26]. The (^32^P)-labeled phosphorylase-*a *was then used as a substrate for PP2A. We also used PP1 as a control. LIS1 inhibited the phosphorylase phosphatase activity of PP2A in a concentration-dependent manner (Fig. 5). In contrast, LIS1 had no effect on the phosphorylase phosphatase activity of PP1 (Fig. 5). When purified peptides containing WD5 or N-terminal domains of LIS1 were used instead of full length LIS1, we observed inhibition of PP2A activity by the WD5 but not the N-terminal domain of LIS1 (Fig. 6A). When the peptides were assayed with PP1, no significant inhibition was observed and the effect of the peptides did not differ at high concentration of the peptides (Fig. 6B). Our results thus indicate that LIS1 might directly inhibit PP2A and that the inhibition of PP2A is likely to be mediated by WD domain(s) of LIS1.

### Binding of Tat to LIS1 does not affect the inhibition of PP2A by LIS1

**Figure 7:**
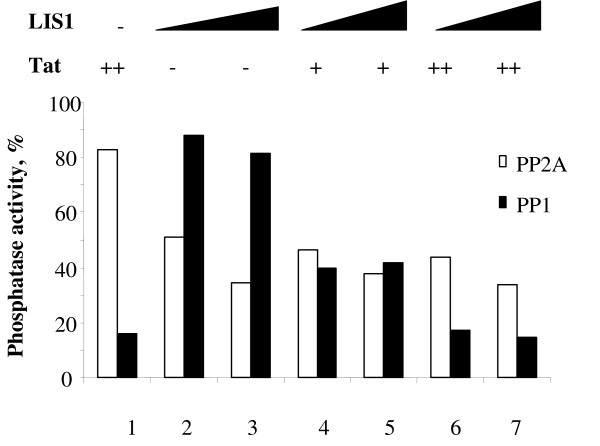
**LIS1 inhibition of PP2A is not altered by Tat**. Phosphatase assay was performed as described in Methods. PP2A (open bars) or PP1 (closed bars) were assayed in the presence of LIS1 and/or Tat. Lane 1, 1 μg of Tat. Lane 2, 0.2 μg of LIS1. Lane 3, 0.4 μg of LIS1. Lane 4, 0.5 μg of Tat and 0.2 μg of LIS1. Lane 5, 0.5 μg of Tat and 0.4 μg of LIS1. Lane 6, 1 μg of Tat and 0.2 μg of LIS1. Lane 7, 1 μg of Tat and 0.4 μg of LIS1.

We next analyzed whether Tat has an effect on the inhibition of PP2A by LIS1. Purified recombinant Tat was added to PP2A or PP1 alone or in combination with LIS1 and phosphorylase phosphatase activity of PP2A or PP1 was assayed. Recombinant Tat was expressed in bacteria and purified by reverse phase chromatography as we previously described [27]. Tat inhibited PP1 but not PP2A (Fig. 7, lane 1). LIS1 inhibited the activity of PP2A but not PP1 (Fig. 7, lanes 2 and 3). Addition of Tat to LIS1 did not change the LIS1 inhibition of PP2A (Fig. 7, lanes 4 to 7). Also addition of LIS1 to Tat did not change the inhibition of PP1 by Tat (Fig. 7, lanes 4 to 7). Thus Tat has no effect on LIS1-mediated inhibition of PP2A.

Taken together, our results show that LIS1 upregulates HIV-1 Tat mediated transcription and that this upregulation could be due to the inhibition or modulation of PP2A activity by LIS1.

## Discussion

Our results presented here show that LIS1 upregulates HIV-1 transcription possibly by inhibiting PP2A. We demonstrate that the WD domains but not the N terminal domain of LIS1 are involved in both upregulation of transcription and PP2A inhibition.

LIS1, a microtubule binding protein [28] regulates microtubule dynamics by interacting with dynein motor, NudC and Dynactin [29,30] and also with Nudel [31]. A yeast homologue of LIS1, NudF associates with NudC to regulate dynein and microtubule dynamics [32,33]. Lissencephaly is a neuronal disease caused by a severe mutation in the LIS1 gene. Interestingly, HIV-1-associated dementia is prevalent in the patients with AIDS. Whether there is a connection between deregulation of LIS1 function and development of dementia is not yet known, but obviously this is an intriguing possibility.

**Figure 8:**
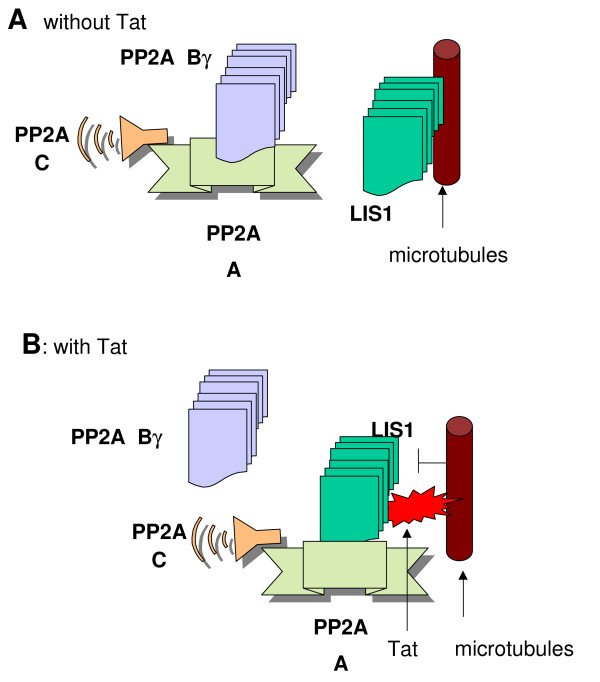
**Proposed mechanism of Tat, LIS1 and PP2Ainteraction**. Binding of Tat to LIS1 may rearrange LIS1 binding to microtubules to allow its interaction with PP2A core enzyme.

We envision a possible mechanism of Tat, LIS1 and PP2A interaction (Fig 8). We propose that LIS1 binds PP2A core enzyme and substitutes the B subunit of PP2A holoenzyme. By substituting the targeting B subunit of PP2A, LIS1 may relocate PP2A to a new substrate and also move it away from its physiological substrate (Fig 8). Tat-dependent HIV-1 transcription requires the activity of CDK9, and CDK9 autophosphorylation was shown to be important for the binding of CDK9/cyclin T1 to TAR RNA [34]. As we have recently shown PP2A dephosphorylates CDK9 and pretreatment of CDK9 with PP2A increases CDK9 autophosphorylation [35]. Thus it is possible that Tat might coordinate CDK9 dephosphorylation by PP2A prior to its recruitment to TAR RNA. Activation of CMV promoter by LIS1 supports this explanation as CMV promoter strongly relies on CDK9 activity [36]. The inhibitory effect of LIS1 on PGK promoter indicates that LIS1 might have a differential effect on cellular promoters. Further studies are needed to analyze the effect of LIS1 on cellular gene expression. We previously showed that Tat interacts with LIS1 *in vitro *and *in vivo *and that LIS1 was part of a larger complex that in addition contained CDK7, cyclin H, MAT1 [10]. It is possible that interaction of Tat with this complex might activate CDK7 and ultimately affect viral gene expression through a direct activation of CDK7 or indirectly through activation of a down stream kinase, CDK2, which we recently showed to be important for HIV-1 transcription [37,38]. As Tat is shuttling between nucleus and cytoplasm, its interaction with LIS1 and CDK7-containing protein complex might allow a temporary activation of the CDK7 activity. Although LIS1 is a cytoplasmic protein, it is often found in the perinuclear area where the initial assembly of the complex containing CDK7 might take place. Finally, LIS1 may also remove PP2A away from IκB, promoting IκB phosphorylation by IκK and release and activation of NF-κB. The liberated NF-κB translocates to the nucleus and increases transcription from responsive promoters including HIV-1 LTR. Taken together, there are number of potential pathways that can be affected by LIS1 interaction with PP2A. Further studies are needed to clarify the detailed mechanism of LIS1 induction of HIV-1 transcription and the role of LIS1 in HIV-1 pathogenesis.

## Methods

### Materials

PP1 was a generous gift from Dr. Bollen (Catholic University of Leuven, Belgium). PP2A was purchased from Upstate (Chicago, IL). Rabbit polyclonal LIS1 antibodies were purchased from Novus Biologicals. Anti-Flag and anti-a-tubulin antibodies were from Sigma.

### Plasmids

The HIV-1 reporter contained HIV-1 LTR (-138 to +82) followed by a nuclear localization signal (NLS) and the LacZ reporter gene (courtesy of Dr. Michael Emmerman, Fred Hutchinson Cancer Institute, Seattle, WA). It expresses NLS-tagged β-galactosidase under the control of HIV-1 LTR (16). The HIV-1 reporter plasmid without TAR contained a deletion of +19 to +87 nucleotides of LTR introduced by restriction digestion with BglII. The Tat expression plasmid was a gift from Dr. Ben Berkhout (University of Amsterdam) (17). The CMV-EGFP cloned into the Adenovirus shuttle vector was a gift from Dr. Marina Jerebtsova (Children's National Medical Center). The SIN vector containing phosphoglycerol kinase (PGK) promoter followed by EGFP [39] was a gift from Dr. John Tisdale (NIDDK, NIH). The PP2A Bγ expression vector [24] was a gift from Dr. Stefan Strack (University of Iowa).

### Expression and purification of WD5 and N terminal of LIS1

The DNA sequences encoding each of the seven WD domains and the amino terminal domain of LIS1 were subcloned into a plasmid carrying T7 promoter upstream of the multiple cloning site and a myc tag and amp^r ^markers. These vectors were created at the laboratory of Dr. Orly Reiner (The Weizmann Institute of Science, Israel) and were kindly given to us. The DNA was transformed into *E. coli BL21 *SI cells. The cells were grown to mid log phase, and synthesis of recombinant proteins was induced by the addition of NaCl to a final concentration of 0.3M for 18 hours according to the recommendation of manufacturer (Invitrogen). The cells were lysed by sonication in a buffer A (10 mM Tris-HCl (pH 7.8), 50 mM NaCl, 1 mM EDTA 1 mM PMSF, 20% glycerol) and inclusion bodies were recovered by centrifugation. Inclusion bodies were dissolved in the buffer A containing additionally 6M urea and proteins were concentrated on microcone spinning tubes (Millipore, Billerica, MA). The recombinant proteins were dialyzed against PBS before usage.

### Cell culture and transfection

Cells were maintained in Dubelco Modified Eagles Medium (DMEM) supplemented with 10% FBS and 0.1%penicillin/streptomycin. HEK293 cells were subcultured 24 hrs prior to transfection to achieve 60% confluence on the day of transfection. Transfections were carried out in 96 well plates and in some experiments in 6 well plates. Transfections were performed by calcium phosphate precipitation as previously described [40].

### Subcloning of pCI-LIS

LIS1 gene was subcloned from pAGA2 vector [10] to pCI-Neo eukaryotic expression vector (Promega, Madison, WI). The pAGA2-LIS1 plasmid was digested with EcoR1 and Sal1 to extract LIS1 fragment. The LIS1-containig DNA fragment was purified on the agarose gel and ligated into the pCI-Neo digested with EcoR1 and Sal1. The resulting plasmid pCI-LIS was checked by restriction digestion with EcoR1 and Sal1 to visualize ligation products on an agarose gel and also by sequencing with T7 and T3 primers.

### β-galactosidase assay

HEK 293 cells transfected with HIV-1 LTR-LacZ and HIV-1 Tat expression vectors were lyzed and the level of transcription from the HIV-1 LTR was determined by measuring the β-galactosidase activity as previously described [40]. Briefly, cells were washed with phosphate-buffered saline (PBS) and lysed for 20 min at room temperature in 50 μl of lysis buffer, containing 20 mM HEPES at pH 7.9, 0.1% Nonidet P-40, and 5 mM EDTA. Subsequently, 100 μl of o-nitrophenyl-β-D-galactopyranoside (ONPG) solution (72 mM Na_2_PO_4 _at pH 7.5, 1 mg/ml ONPG, 12 mM MgCl_2_, 180 mM 2-mercaptoethanol) was added and incubated at room temperature until a yellow color developed. The reaction was stopped by addition of 100 μl of 1 M Na_2_CO_3_. The 96-well plates were analyzed in a micro plate reader at 414 nm (Lab Systems Multiscan MS). The β-galactotosidase units were calculated using a linear graph plotted from optical density (OD) readings of the standard.

### Florescence measurement

293T cells transfected in 96-well plate were lysed in 50 μl of lysis buffer per well (20 mM HEPES at pH 7.9, 0.1% Nonidet P-40, and 5 mM EDTA), then supplemented with 150 μl of PBS and transferred to fluorescent-compatible 96-well plate. The GFP fluorescence was measured at 480 nm excitation and 510 nm emission on Luminescence Spectrometer LS50B (Perkin-Elmer) equipped with the robotic 96-well scanner.

### Western blot

Cells transfected with various vectors were washed 3 times with PBS and then lyzed with lysis buffer containing 50 mM Tris-HCl (pH 7.5), 0.5 M NaCl, 1% NP-40, 0.1% SDS and protease inhibitor cocktail (Sigma). The control cell extracts were prepared from mock-transfected 293T cells. About 5 μg whole cell extract proteins were resuspended in a 30 μl of 1× SDS loading buffer (4% SDS, 10% glycerol, 5% 2-mecarpthaethanol, 0.002% bromophenol blue) and heated at 90°C for 3 minutes. The proteins were resolved on 10% SDS Polyacrylamide gel electrophoresis (PAGE) and immunoblotted with anti-LIS1 or anti tubulin antibodies

### Phosphatase assay

Phosphorylase-*a *was prepared as previously described [26]. Approximately 0.2 nmol of phosphorylase-*a *was used as a substrate for PP1 or PP2A. The phosphorylase phosphatase assay was carried out for 10 min in a buffer containing 50 mM glycylglycine at pH 7.4, 0.5 mM dithiothreitol, and 5 mM β-mercaptoethanol as described [41]. Where indicated, prior to the phosphorylase phosphatase assay, the samples were trypsinized to generate free, active catalytic subunit of PP1.

## Competing interests

The author(s) declare that they have no competing interests.

## Authors' contributions

NE created LIS1 expression vector, purified WD5 and N-terminal peptides, and conducted transfection experiments and some of the phosphatase inhibition assays. TA prepared phosphorylase-a and helped to conduct phosphatase assays. WT participated in the design and discussion of the study. SN performed general control and coordination of the study. All authors read and approved the manuscript.
